# An integrated biophysical fragment screening approach identifies novel binders of the CD28 immune receptor

**DOI:** 10.1016/j.bbrep.2026.102542

**Published:** 2026-03-07

**Authors:** Laura Calvo-Barreiro, Hossam Nada, Moustafa T. Gabr

**Affiliations:** Department of Radiology, Molecular Imaging Innovations Institute (MI3), Weill Cornell Medicine, New York, NY, 10065, USA

## Abstract

CD28 is an essential costimulatory receptor required for full T cell activation and its dysregulation contributes to multiple immune-mediated pathologies. Despite its central immunological role, CD28 remains largely unexplored as a target for small-molecule modulation, primarily due to the shallow and large interface of its ligand-binding site. Here, we applied a fragment-based high-throughput screening (HTS) strategy to identify low molecular weight chemotypes capable of engaging with human CD28. A 3200-member library composed of structurally diverse fragments, enriched for scaffolds designed to target protein-protein interaction (PPI) interfaces, was screened in single-dose format using temperature-related intensity change (TRIC) technology, yielding 36 primary hits (1.13% hit rate). Follow-up surface plasmon resonance (SPR) validation confirmed two fragments as direct CD28 binders. Molecular docking analysis revealed a plausible binding orientation for PPIF3 within the CD28 extracellular domain, suggesting potential interaction hotspots that may be exploited during future optimization. Together, these findings provide the first demonstration that fragment-based screening can successfully identify chemotypes capable of engaging with the CD28 PPI interface. This work establishes a scalable, biophysics-driven workflow for CD28 ligand discovery and lays the foundation for subsequent hit-to-lead development of small molecule CD28 modulators.

## Highlights

• Fragment-based TRIC-SPR screening identifies direct CD28 binders.
• First fragment hits reported for the CD28 immune receptor.
• PPIF3 shows measurable affinity and a unique binding mode.
• Fragments engage interface features not used by previous small molecules.
• Provides new starting points for CD28-focused drug discovery.

## 1 Introduction

The costimulatory receptor CD28 is a central regulator of T-cell activation, survival, and homeostasis. By integrating signals from antigen-presenting cells through binding to its ligands (CD80/CD86), CD28 ensures proper immune priming and effector responses. Dysregulated CD28 signaling contributes to multiple immune-mediated pathologies, including autoimmunity, transplant rejection, and aberrant T-cell activation in cancer [1]. Given its pivotal immunological role and tractable extracellular domain, CD28 represents an attractive therapeutic target for selective modulation of T-cell function. Despite this importance, CD28 has remained largely underexplored as a small-molecule target due to the inherent challenges of its ligand-binding surface. Structural studies have shown that CD28 recognizes CD80/CD86 through a shallow, relatively flat protein-protein interaction (PPI) interface, which lacks deep pockets commonly exploited by drug-like molecules [1]. As a result, conventional screening approaches often struggle to identify tractable chemical matter for this receptor.

Over the past decade, small-molecule approaches have gained momentum as complementary strategies to biologics for targeting immune checkpoints [2]. Traditional high-throughput screening (HTS) can identify potent inhibitors or modulators, but the complexity and low druggability of PPIs make the discovery of novel chemotypes challenging. Fragment-based drug discovery (FBDD) has emerged as a powerful alternative, enabling the detection of low-molecular-weight ligands that engage shallow binding surfaces with high ligand efficiency. These fragment hits can be elaborated into more potent, selective scaffolds using medicinal chemistry and structure-based optimization [3]. FBDD is particularly well suited for receptors like CD28 because fragments are capable of probing sparse or topologically feature-poor surfaces, revealing interaction hot spots that may remain undetected by traditional screening chemotypes. However, the identification of fragment binders at challenging PPI interfaces requires biophysical methods with the sensitivity to detect weak interactions.

In this work, we applied a fragment-based screening strategy to explore the chemical tractability of the CD28 extracellular domain using sensitive biophysical detection methods. TRIC technology enables the screening of low molecular-weight (MW) compounds thanks to its mass-independent technology in a solution-based 384-well plate format. This combination of features allows our biophysical screen to closely approximate physiological conditions, while avoiding the loss of sensitivity that can occur on other platforms when working with very small molecules (MW ranging between 190 Da and 460 Da). By integrating TRIC-based primary screening with orthogonal SPR validation and computational modeling, we sought to determine whether fragment-sized chemotypes could directly engage this historically challenging PPI interface. This study establishes a scalable biophysical workflow for CD28 ligand discovery and provides a foundation for future hit-to-lead optimization efforts.

## 2 Methods

### 2.1 Screened library

A subset of 3200 compounds from the PPI Fragment Library (Cat. # PPIF-3600, Enamine, Kyiv, Ukraine) was selected as the compound library targeting the human CD28 protein. All compounds were contained in 384-well plates and dissolved in DMSO at a concentration equal to 10 mM. Libraries were stored at −80 °C upon arrival and until use.

Following HTS, newly lyophilized material for each selected primary hit was obtained from Enamine. These compounds were reconstituted in DMSO at a concentration of 50 mM and stored at −30 °C until use.

### 2.2 Dianthus high-throughput affinity screening

Affinity screening was performed using the Dianthus NT.23 Pico platform (NanoTemper Technologies, München, Germany) as previously described [4]. Briefly, His-tagged human CD28 protein (Cat. # CD8-H52H3, Acro Biosystems, Newark, DE, USA) was labeled with Monolith His-Tag Labeling Kit RED-tris-NTA 2nd Generation (Cat. # MO-L018, NanoTemper Technologies) and incubated with test compounds or controls. Fluorescence changes were measured via temperature-related intensity change (TRIC) to assess binding when compared to the negative control. Compounds showing interaction with the dye alone were excluded. Confirmed binders were selected for confirmatory testing using orthogonal validation by SPR.

### 2.3 Biacore™ 8K single-dose screening

Single-dose binding assays (200 μM) were performed using the Biacore™ 8K system (Cytiva, Marlborough, MA, USA) at 25 °C, following previously published protocol and data analysis [5]. Biotinylated human CD28 (Cat. # CD8-H82E5, Acro Biosystems) was immobilized using the Biotin CAPture Kit (Cat. # BR100669, Cytiva), and candidate compounds were injected in 1x PBS-P buffer with 2% DMSO. Anti-CD28 antibody served as a positive control, and solvent corrections were applied to account for DMSO-related refractive index changes. Data acquisition and analysis were conducted using Biacore™ 8K Control and Insight Evaluation Software, respectively, to identify and rank primary hits.

### 2.4 SPR-based binding affinity screening

Binding kinetics of selected hits were evaluated using a multi-cycle assay on the Biacore™ 8K system (Cytiva) at 25 °C, following previously published procedures [5]. Briefly, biotinylated CD28 was immobilized using the Biotin CAPture Kit, and analytes were injected at ascending concentrations (ranging from 43.9 μM up to 500 μM) in 1x PBS-P buffer with 2% DMSO. All sensorgrams were double-referenced by subtracting the response from a reference flow cell and from blank buffer injections. Solvent correction curves spanning the relevant DMSO concentration range were applied to minimize bulk refractive-index contributions. These procedures were implemented to ensure accurate response normalization prior to affinity determination. Sensorgrams were analyzed using Biacore™ Insight Evaluation Software to determine steady-state affinities.

### 2.5 Molecular docking

The molecular docking employed in this study followed the same computational methodology as previously described in our recently published work [5,6].

## 3 Results and discussion

### 3.1 Fragment screening of CD28 using TRIC technology

Building on the optimized TRIC workflow previously developed for CD28 [4], we applied the same assay design and criteria to screen a subset of the Enamine PPI Fragment Library [7]. This screening collection contains 3200 structurally diverse fragments specifically engineered to explore PPI interfaces and enriched in substructures known to interact with hot-spot residues. Because CD28 engages with its ligands (CD80/CD86) through a shallow, relatively flat interface, this library provided an appropriate source of low MW chemotypes with physicochemical properties (balanced polarity, scaffold diversity, and PPI-mimicking features) well suited for detecting weak but specific binders [7].

**Fig. 1:**
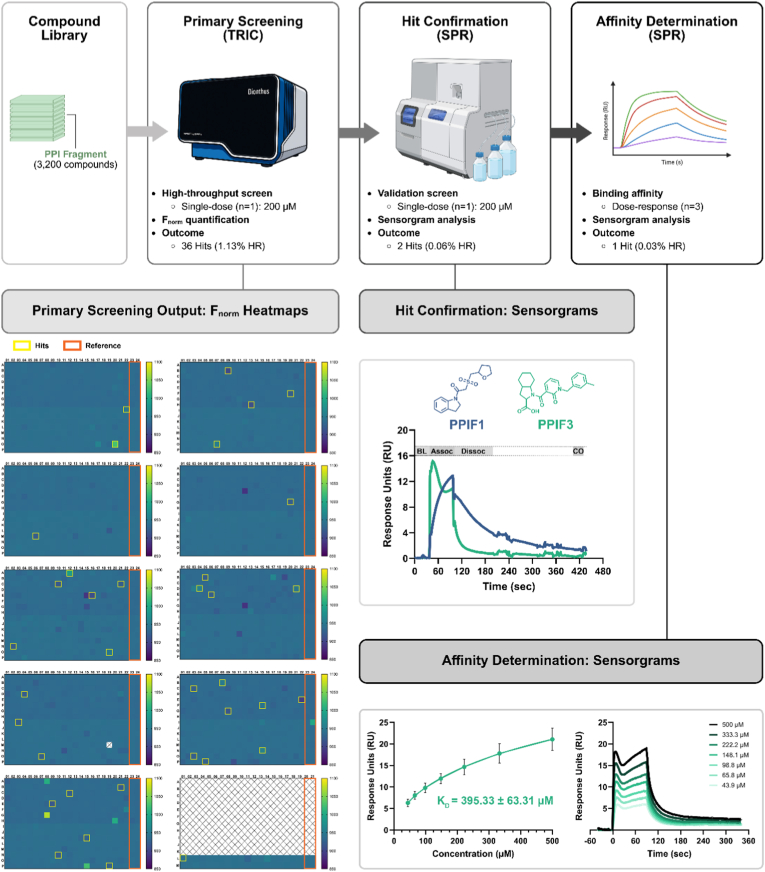
**Workflow and results of the primary screening, hit confirmation, and affinity determination for CD28-targeted PPI fragments.** Schematic overview of the screening funnel integrating TRIC-based primary screening with SPR-based hit validation and affinity characterization. A 3200-member PPI Fragment library was screened in single-dose format (200 μM), yielding 36 primary hits (1.13% HR) based on F_norm_ output. 384-well plate heatmaps illustrate F_norm_ distributions and highlight wells exceeding hit thresholds (yellow boxes) and reference wells included as experimental negative controls (orange wells). Wells highlighted in yellow represent fragments meeting predefined ΔF_norm_ statistical thresholds and passing signal-quality filters. Wells with visually apparent signal deviations but not boxed were excluded due to failing ΔF_norm_ cutoff criteria or exhibiting autofluorescence, quenching, or irregular trace behavior. Selected primary hits were subjected to SPR validation using a single-dose injection (200 μM), resulting in two confirmed binders (0.06% HR) with clear association and dissociation phases. Representative sensorgrams for the two validated hits, PPIF1 and PPIF3, are shown. Following a stable baseline (BL), each 200 μM fragment solution was injected over the sensor surface for 60 s (association phase, Assoc), and the resulting response (RU) relative to baseline was recorded. A 100 s dissociation phase (Dissoc) allowed disengagement from the active flow cell, after which a carryover control (CO) step was performed by flowing additional running buffer over both flow cells. Subsequent dose-response SPR analysis identified one fragment with quantifiable binding affinity (K_D_ = 395.33 ± 63.31 μM; 0.03% overall HR). Fitted steady-state curves (three independent experiments, mean ± SD) and representative sensorgrams (one independent experiment) are shown. Sensorgrams shown are double-referenced and solvent-corrected. Minor bulk-associated signal features during injection are characteristic of fragment-level interactions at high analyte concentrations and do not preclude steady-state response analysis. Abbreviations: HR: Hit Rate, PPI: Protein-Protein Interaction, SPR: Surface Plasmon Resonance, TRIC: Temperature-Related Intensity Change.

Using freshly labeled CD28 protein and the optimized PBS-based buffer, we screened all 3200 fragments at a final concentration of 200 μM. Each plate included two reference columns containing only assay buffer supplemented with DMSO, serving as negative controls (Fig. 1, Primary Screening Output). As in our previously validated HTS workflow, a single F_norm_ value was obtained for each fragment and compared to the distribution of reference wells to calculate the ΔF_norm_ response. F_norm_ values were calculated as the normalized fluorescence response following temperature perturbation relative to baseline fluorescence intensity. ΔF_norm_ was defined as the difference between the F_norm_ value of each compound-containing well and the mean F_norm_ of the DMSO reference wells on the same plate. Hit selection was based on signed positive ΔF_norm_ values exceeding three times the standard deviation of the DMSO reference wells. To further reduce false positives, an additional fixed ΔF_norm_ threshold of 10 units was applied. Absolute ΔF_norm_ values were not used for hit calling [4]. Although certain wells in Fig. 1 display visually noticeable signal deviations, not all were advanced as hits. Wells exhibiting strong negative ΔF_norm_ values were excluded, as such responses typically reflect fluorescence quenching or signal interference rather than specific binding events. Additionally, wells showing autofluorescence, irregular TRIC trace morphology, unstable baselines, or non-reproducible signal behavior were removed during manual triage prior to SPR validation. Only wells meeting all predefined statistical and quality-control criteria are boxed as hits in Fig. 1. After triage, 36 fragments remained as primary hits and were cherry-picked for follow-up testing (Table 1; Fig. 1, Primary Screening Output).

**Table 1:** **Primary fragment hits identified from the TRIC-based high-throughput screen and validated using SPR.** The table summarizes biophysical parameters for all compounds progressing from the TRIC primary screen to SPR follow-up validation. For each analyte, the molecular weight (MW), analyte binding level (measured at the end of the SPR association phase), immobilized ligand level, calculated theoretical R_max_, and resulting Level of Occupancy (LO) are reported. Compounds were advanced to affinity characterization if they displayed an LO between 50 and 100% and were not flagged as non-specific binders (NSB). All measurements were obtained using biotinylated human CD28 ectodomain (70 kDa) as the immobilized ligand. Abbreviations: LO, level of occupancy; MW, molecular weight; NSB, non-specific binder; RU, response units.

Analyte	Analyte MW (Da)	Analyte Binding Level (RU)	Immobilized Ligand Level (RU)*	R_max_ (RU)	LO (%)	Curve Marker
PPIF1	309.4	12.1	1646.1	14.55	82.95	
PPIF2	279.2	10.4	1646.1	13.13	78.93	NSB
PPIF3	394.5	11.6	1646.1	18.55	62.37	
PPIF4	259.3	1.4	1682.8	12.47	11.16	
PPIF5	354.8	1.1	1646.1	16.69	6.577	NSB
PPIF6	337.3	0.6	1703.3	16.41	3.844	
PPIF7	361.8	0.2	1646.1	17.02	1.188	
PPIF8	371.5	−0.3	1703.3	18.08	−1.61	
PPIF9	369.5	−0.3	1646.1	17.38	−1.858	
PPIF10	359.4	−0.8	1703.3	17.49	−4.417	
PPIF11	372.8	−0.8	1703.3	18.14	−4.584	
PPIF12	344.8	−0.8	1682.8	16.58	−4.76	
PPIF13	278.8	−0.8	1682.8	13.4	−5.806	
PPIF14	373.4	−1.1	1703.3	18.17	−5.816	
PPIF15	342.4	−1.3	1646.1	16.1	−8.323	
PPIF16	376.3	−1.8	1682.8	18.09	−9.751	
PPIF17	364.9	−1.7	1646.1	17.16	−9.894	
PPIF18	265.7	−1.3	1703.3	12.93	−10.16	
PPIF19	352.4	−2.1	1682.8	16.94	−12.52	
PPIF20	359.8	−2.8	1703.3	17.51	−16.04	
PPIF21	379.5	−3.4	1703.3	18.47	−18.52	
PPIF22	349.4	−3.3	1682.8	16.8	−19.82	
PPIF23	231.3	−2.4	1703.3	11.26	−21.28	
PPIF24	397.4	−4.2	1646.1	18.69	−22.58	
PPIF25	312.4	−5	1682.8	15.02	−33.21	
PPIF26	349.4	−6	1682.8	16.8	−35.6	NSB
PPIF27	348.4	−6.5	1703.3	16.96	−38.37	
PPIF28	385.5	−7.3	1646.1	18.13	−40.28	NSB
PPIF29	347.4	−8	1703.3	16.91	−47.27	NSB
PPIF30	362.4	−9.2	1646.1	17.04	−54.05	NSB
PPIF31	345.4	−12.8	1682.8	16.61	−76.95	NSB
PPIF32	348.8	−17	1703.3	16.97	−99.91	
PPIF33	398.5	−20.6	1682.8	19.16	−107.5	NSB
PPIF34	367.4	−24.5	1682.8	17.66	−138.6	
PPIF35	349.4	−23.8	1682.8	16.8	−141.6	NSB

### 3.2 Biophysical validation of hits using SPR

Following TRIC-based hit selection, commercially available primary fragments (35 out of the initial 36 candidates) were evaluated using SPR to assess direct binding to immobilized CD28 (Table 1). We adopted an SPR workflow aligned with the previously published CD28 HTS platform, screening fragments based on level of occupancy (LO), response magnitude, and dissociation kinetics [5]. Hits were considered validated only when they achieved LO > 50%, displayed clean association/dissociation profiles, and passed all specificity filters, including absence of non-specific binding to the reference flow-cell and lack of drift or non-dissociating behavior. Compounds exhibiting non-dissociating responses, signal drift, baseline instability, or disproportionate responses relative to theoretical Rmax were classified as non-specific binders and excluded from further analysis. This filtering step was implemented to minimize the contribution of aggregation-related or colloidal artifacts. This strict gate mirrors the validated criteria reported in the original study and ensures high confidence in true positives. Given the low-affinity fragment-level interactions observed, affinity values were determined using steady-state response analysis rather than kinetic rate fitting. This approach is appropriate when full equilibrium plateaus are not fully developed within the injection window at high micromolar analyte concentrations.

Using this orthogonal validation approach, two fragments exhibited measurable binding responses at 200 μM in a single-dose experiment (Fig. 1, Hit Confirmation). For one of them, PPIF3, a full concentration series (ranging 500 to 43.9 μM) enabled determination of an affinity constant, yielding a K_D_ of 395.33 ± 63.31 μM, consistent with fragment-like binding (Fig. 1, Affinity Determination) [8]. Equilibrium approximation was assessed by evaluating response stabilization during the final phase of injection, reproducibility across independent experiments, and consistency of fitted steady-state models. The reported K_D_ therefore represents an apparent steady-state affinity under near-equilibrium conditions. The second hit, PPIF1, showed clear binding but did not reach saturation within the tested concentration range, preventing reliable K_D_ estimation. As expected for fragment-sized analytes evaluated at high micromolar concentrations, transient bulk-related signal contributions during injection were observed despite solvent correction; however, responses were reproducible across independent experiments and displayed concentration-dependent increases consistent with specific target engagement. Overall, SPR validation not only confirmed direct binding but also provided kinetic insights that strengthened prioritization of the selected fragments for downstream characterization. The validated fragments displayed reproducible concentration-dependent responses with observable dissociation phases, behavior inconsistent with persistent surface retention typically associated with colloidal aggregation.

### 3.3 Docking analysis to explore potential binding interactions

The costimulatory receptor CD28 and its inhibitory counterpart CTLA-4 engage B7 family ligands (CD80/CD86) through a shared binding mechanism. Crystallographic analyses have shown that their primary ligand-binding region resides within a conserved segment encompassing residues 99-104^9^. Mutagenesis studies further confirmed the functional importance of this site, as substitutions within this sequence cause a marked (>90%) reduction in ligand-binding affinity. Despite its critical role in receptor-ligand recognition, the CD28 binding interface poses major challenges for small-molecule drug design. Structural analysis of the CD28^−^CD80 complex (PDB ID: 1YJD [9]) reveals a relatively flat and extended interaction surface, lacking the deep, well-defined pockets typically required for high-affinity small-molecule binding. Such shallow protein-protein interfaces are inherently difficult to target with drug-like compounds because they offer limited opportunities for specific and stable molecular interactions [5].

To gain preliminary insight into potential molecular mechanisms underlying the biological activity of PPIF3, we performed computational docking studies to explore possible interaction modes with the CD28 receptor. Molecular docking was performed using Schrödinger 2024.3 (Glide module) with default parameters (standard precision mode) against the CD28 crystal structure. The receptor structure was prepared using the Protein Preparation Wizard, including hydrogen addition, bond order assignment, and restrained energy minimization. The docking grid was centered on the CD80/CD86 binding interface, encompassing the known ligand-binding region. The top-ranked pose was selected based on the GlideScore docking score and is presented here as a hypothesis for subsequent experimental validation.

**Fig. 2:**
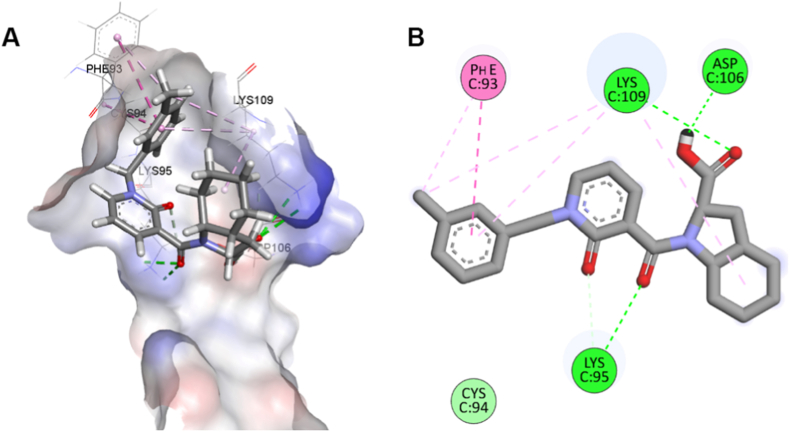
**Molecular docking analysis of PPIF3 binding to CD28.** (A) Three-dimensional representation of PPIF3 bound to the CD28 receptor. (B) Two-dimensional interaction diagram of PPIF3 with CD28.

The computational analysis suggests that PPIF3 may adopt a binding orientation within the CD28 ligand-binding region, though this differs spatially from the binding modes of previously reported ligands (Fig. 2A and B). Analysis of the predicted docking pose indicates potential hydrogen bonding interactions with Lys95, Lys109, and Asp106, which are known to be critical residues for B7 ligand recognition. Additionally, Phe93 may contribute to complex stabilization through hydrophobic π-π stacking interactions with the aromatic core of PPIF3, collectively suggesting a well-defined and energetically favorable binding mode (Fig. 2B). It should be noted that these computational predictions remain hypothetical and require experimental validation.

Several previously reported CD28-targeted small molecules, including those identified through virtual screening, mass spectrometry, and TRIC-based HTS workflows [[4], [5], [6],^10^] belong predominantly to higher molecular weight drug-like scaffolds. These chemotypes generally rely on multi-point interactions distributed across the relatively flat CD28-B7 interface.

Because such compounds occupy a larger surface footprint, their engagement often depends on combining hydrophobic interactions with extended π-systems or hydrogen-bonding networks spanning multiple secondary structural elements. This recognition pattern aligns with expectations for PPI-directed inhibitors, which often require considerable surface area coverage to compensate for the absence of deep, pocket-like cavities.

In contrast, the fragment hit identified here, PPIF3, is substantially smaller and exhibits a more compact pharmacophore. Our computational docking analysis suggests that PPIF3 may not mimic the canonical binding profiles observed for previously described small molecules. As a working hypothesis, PPIF3 appears to localize within a more restricted microenvironment of the CD28 interface, forming a focused cluster of predicted interactions with Lys95, Asp106, Lys109, and Phe93. These residues are known to lie within or adjacent to the CD80/CD86 contact region, yet the specific spatial configuration suggested for PPIF3 differs from the surface-spanning arrangements formed by larger ligands. If validated experimentally, this would suggest that PPIF3 is not merely a downsized version of existing chemotypes, but rather a fragment that exploits an orthogonal interaction motif within the CD28 extracellular domain.

The computational prediction of such an alternative binding orientation would be noteworthy for two reasons if confirmed experimentally. First, it suggests that the CD28 interface, despite its shallow and historically challenging topology, may contain localized interaction hot spots capable of supporting fragment-sized ligands. Second, it highlights the ability of fragment-based approaches to uncover binding modes that may not be accessible through conventional HTS libraries. While larger molecules often rely on extensive surface coverage across the interface, fragments can instead anchor within subtle topological features, forming efficient and focused interactions that might otherwise remain undetected.

Although direct comparison between fragment hits and drug-like CD28 ligands must be interpreted cautiously, given differences in affinity, size, and screening modality, the hypothesized distinct binding mode for PPIF3 underscores the complementary nature of fragment-based approaches. Fragments such as PPIF3 can potentially reveal previously unappreciated binding vectors, identify incipient pockets or transient subpockets, and provide medicinal chemistry starting points that are chemically tractable and structurally divergent from prior scaffolds. Collectively, these findings indicate that biophysical fragment screening can expand the accessible chemical space for CD28 modulation and may ultimately enable the rational evolution of fragments into higher-affinity ligands with unique mechanisms of action, pending experimental validation of the predicted binding interactions. Unlike our earlier CD28 discovery efforts, which prioritized functional inhibition and drug-like scaffolds, this study focuses on fragment-level engagement as a strategy to uncover minimal interaction motifs within the CD28 interface. The identification of fragment binders expands the accessible chemical starting points for CD28 modulation and complements previously reported inhibitor scaffolds.

## 4 Conclusions

We have conducted the largest HTS to date using fragment-based approaches targeting the CD28 immune receptor with biophysical techniques. Our previous CD28-targeted campaigns primarily focused on drug-like libraries and structure-based virtual screening strategies to identify higher-molecular-weight inhibitors with functional activity. In contrast, the present study specifically investigates whether fragment-sized chemotypes can directly engage the CD28 extracellular domain, thereby probing the tractability of this shallow PPI interface at an earlier stage of chemical space exploration. From a 3200-fragment library, we identified and validated an efficient fragment that binds to the protein surface. The overall low hit rate of 0.03% underscores the challenges inherent to this class of targets which has also been observed in previous campaigns [[4], [5], [6],^10^]. Thus, our findings highlight both the structural difficulties and the opportunities in modulating the CD28-B7 costimulatory axis with small molecules.

Although the CD28 ligand-binding interface is shallow and lacks well-defined pockets, our molecular docking studies demonstrate that selective fragment engagement is achievable. PPIF3 adopts a distinct and energetically favorable binding mode within the CD28 interface. Its ability to form stabilizing hydrogen bonds with key recognition residues (Lys95, Asp106, Lys109) and hydrophobic π-π interactions with Phe93 supports a plausible molecular mechanism for its observed micromolar activity. These results provide a structural rationale for fragment binding at this challenging interface and lay the foundation for structure-guided optimization toward higher-affinity CD28 modulators.

## CRediT authorship contribution statement

**Laura Calvo-Barreiro:** Conceptualization, Data curation, Formal analysis, Investigation, Methodology, Project administration, Visualization, Writing – original draft. **Hossam Nada:** Investigation, Methodology, Writing – original draft. **Moustafa T. Gabr:** Funding acquisition, Supervision, Writing – review & editing.

## Declaration of competing interest

The authors declare that they have no known competing financial interests or personal relationships that could have appeared to influence the work reported in this paper.

## Data Availability

Data will be made available on request.
